# Gene silencing of IL-12 in dendritic cells inhibits autoimmune arthritis

**DOI:** 10.1186/1479-5876-10-19

**Published:** 2012-01-31

**Authors:** Rong Li, Xiufen Zheng, Igor Popov, Xusheng Zhang, Hongmei Wang, Motohiko Suzuki, Rosalia De Necochea-Campion, Peter W French, Di Chen, Leo Siu, David Koos, Robert D Inman, Wei-Ping Min

**Affiliations:** 1 Institute of Immunomodulation and Immunotherapy, Nanchang University Medical School, Nanchang, China; 2 Departments of Surgery, Pathology, and Oncology, University of Western Ontario, London, Canada; 3 Medistem Inc, San Diego, CA; 4 Benitec Biopharma Limited, Sydney, NSW, Australia; 5 Entest BioMedical, San Diego, CA; 6 Division of Rheumatology, Department of Medicine, Toronto Western Hospital, University Health Network, Toronto, Canada; 7 University Hospital C9-136, 339 Windermere Road, London, Ontario, N6A 5A5, Canada

**Keywords:** shRNA, IL-12, Dendritic cells, Autoimmunity, Collagen-induced arthritis

## Abstract

**Background:**

We have previously demonstrated that immune modulation can be accomplished by administration of gene silenced dendritic cells (DC) using siRNA. In this study, we demonstrate the therapeutic utilization of shRNA-modified DC as an antigen-specific tolerogenic vaccine strategy for autoimmune arthritis.

**Methods:**

A shRNA that specifically targets IL-12 p35 was designed and cloned into a plasmid vectors (IL-12 shRNA). Bone marrow-derived DC from DBA/1 mice were transfected with the IL-12 shRNA construct in vitro. Mice with collagen II (CII)-induced arthritis (CIA) were treated with the modified DCs expressing the shRNA. Recall response and disease progression were assessed.

**Results:**

After gene silencing of IL-12 in DC, DC were shown to selectively inhibit T cell proliferation on recall responses and in an MLR. In murine CIA, we demonstrated that administration of IL-12 shRNA-expressing DC that were pulsed with CII inhibited progression of arthritis. The therapeutic effects were evidenced by decreased clinical scores, inhibition of inflammatory cell infiltration in the joint, and suppression of T cell and B cell responses to CII.

**Conclusion:**

We demonstrate a novel tolerance-inducing protocol for the treatment of autoimmune inflammatory joint disease in which the target antigen is known, utilizing DNA-directed RNA interference.

## Background

Rheumatoid Arthritis (RA) is a chronic autoimmune condition characterized by non-specific, usually symmetric inflammation of the peripheral joints, resulting in progressive destruction of articular and periarticular structures. One of the hallmark pathologies of RA is thickening and swelling of synovial tissue, primarily as a result of T cell production of inflammatory factors [1,2]. Up to 50% of the infiltrating leukocytes in the synovium are T-lymphocytes, primarily CD4^+ ^T cells with an activated/memory phenotype [3-5], expressing a Th1 bias [5,6]. Clinical treatment of RA involves initiating Disease Modifying Anti-Rheumatic Drug (DMARD) therapy early following diagnosis with subsequent optimization of drug therapy in order to have a greater beneficial impact on disease outcome [7]. DMARDs are antigen-nonspecific in their activities and include known immune suppressants such as methotrexate, leflunomide, hydroxychloroquine, sulfasalazine, and corticosteroids. The introduction of "biological DMARDs" such as Embrel and Remicade led to a major improvement in quality of life of RA patients, however these drugs are limited by cost, non-cure of the disease, and adverse effects such as heightened risk of infection [8,9].

Despite promising animal data, to date, antigen-specific treatments of RA have not been clinically successful. While approaches such as intravenous immunoglobulin [10], oral tolerance [11,12], and tolerogenic peptide therapy [13] have demonstrated promising results in various models, clinical trials have yielded results that are mediocre at best. Dendritic cell (DC) therapy is considered one of the most potent means of antigen-specifically modulating an immune response given the innate propensity of DC to either activate or inhibit adaptive immune responses [14-17]. The recent FDA approval of Provenge as an antigen-specific immunotherapy for prostate cancer attests to the ability of this approach to be translated clinically [18]. Although exceptions exist, generally speaking, in immature states, DC act primarily as tolerogenic cells, caused deviation of Th1 immunity, as well as generation of T regulatory cells [19,20], whereas mature DC are immune stimulatory. We have previously applied these findings in the animal model of RA, collagen induced arthritis (CIA) to demonstrate that DC made immature by treatment with a synthetic RelB inhibitor prevented disease progression [21]. These findings were confirmed in subsequent studies in which we generated "artificially immature" DC using siRNA to silence the markers of maturation, CD40, CD80, and CD86. When these DC were pulsed with collagen II, the autoantigen implicated in CIA, we observed regression of disease [22,23]. Given that T cell activation involves not only cell surface costimulatory molecules but also cytokines, we chose to examine whether silencing of the cytokine IL-12 on DC would also induce a pro-tolerogenic activity.

The cytokine IL-12 is a soluble factor used by the DC to guide differentiation of naïve T cells into a Th1, cytotoxic/inflammatory state [24-26]. Several studies suggest that IL-12 is associated with autoimmunity in a pathologies such as arthritis [27,28], diabetes [29,30], multiple sclerosis [31,32], and thyroiditis [33,34]. Therefore, a method of selectively inhibiting the IL-12 production at the level of the DC may be an ideal mechanism of immunotherapy for autoimmune diseases. Supporting the importance of IL-12 in DC mediated immune modulation, we have previously demonstrated that siRNA-mediated silencing of the IL-12p35 gene on DC causes immune deviation on recall response towards a Th2-like profile [35]. In the current study we silenced the IL-12p35 gene on DC that were pulsed with collagen II protein. We demonstrated that administration of this antigen specific "tolerogenic vaccine" was capable of inducing a Th2-biased recall response, as well as suppression of pathology in the CIA model. These findings may be supportive of future clinical development using IL-12p35 silenced antigen-pulsed DC.

## Methods

### Animals

Male DBA/1 LacJ and BALB/c mice (The Jackson Laboratories, Bar Harbor, ME), 5 weeks of age, were kept in filter-top cages in the Animal Care and Veterinary Services Facility at the University of Western Ontario according to the Canadian Council for Animal Care Guidelines. Mice were fed by food and water and allowed to settle for 2 weeks before initiation of experiments, which had ethical approval from the university review board.

### CIA model

DBA/1 LacJ mice, 7 weeks of age, were intradermally immunized (Day 0) at several sites into the base of the tail with 200 μg of bovine type II collagen (CII) (Sigma-Aldrich, St. Louis, MO) dissolved in 100 μl of 0.05 M acetic acid and mixed with an equal volume of complete Freund's adjuvant (CFA) (Sigma). CII was dissolved at a concentration of 2 mg/ml by stirring overnight at 4°C. On day 21 after priming, the mice received an intraperitoneal booster injection with 200 μg of CII in the equal volume (100 μl) of PBS. Mice were examined visually three times per week for the appearance of arthritis in the peripheral joints, and arthritis score index for disease severity was given as follows: 0 - no evidence of erythema and swelling; 1 - erythema and mild swelling confined to the mid-foot (tarsals) or ankle joint; 2 - erythema and mild swelling extending from the ankle to the mid-foot; 3 - erythema and moderate swelling extending from the ankle to the metatarsal joints; 4 - erythema and severe swelling encompass the ankle, foot, and digits. Scoring was done by two independent observers, without knowledge of the experimental and control groups.

### DC cultures

At Day 0, bone marrow cells were flushed from the femurs and tibias of DBA/1 LacJ mice, washed and cultured in 6-well plates (Corning, NY) at 4 × 10^6 ^cells/well in 4 ml of complete medium (RPMI 1640 supplemented with 2 mM L-glutamine, 100 U/ml penicillin, 100 μg of streptomycin, 50 μM 2-ME, and 10% FCS (all from Life Technologies, Ontario, Canada) supplemented with recombinant GM-CSF (10 ng/ml; PeproTech, Rocky Hill, NJ) and recombinant mouse IL-4 (10 ng/ml; PeproTech). All cultures were incubated at 37°C in 5% humidified CO_2_. Non-adherent cells were removed after 48 h of culture (Day 2) and fresh medium was added every 48 h.

### shRNA expressing vectors and transfection

siRNA sequences were selected according to the method of Elbashir SM et al. [36]. The siRNA sequence specific for IL-12p35 (AACCUGCUGAAGACCACAGAU) was selected and cloned into Psilencer 3.1 vector (Ambion, Austin, TX) which expresses short hairpin RNA (shRNA) under the control of the mouse U6 promoter, using the method described by the supplier of the vector. IL-12 shRNA was sequenced and prepared in a large scale for in vitro and in vivo study. Gene silencing was examined with DC. DC were generated from bone marrow progenitor cells as previously described [35]. Transfection of DC was conducted as described previously [36]. 24 h before transfection (day 4), DC were plated to be 60-90% confluent on the day of transfection. On day 5, 2 μg of IL-12 shRNA and 3 μl of GenePORTER reagent (Gene Therapy Systems, San Diego, CA) were separately diluted with serum-free medium RPMI 1640 using 1/2 of the transfection volume (125 μl). The diluted DNA was added to the diluted GenePORTER reagent, mixed rapidly and incubated in total volume of 250 μl of the medium at room temperature for 45 min. The culture medium from the DC was aspirated, and the DNA-GenePORTER mixture was added carefully to the DC. Mock controls were transfected with 3 μl of the GenePORTER reagent alone. After 4-h incubation, an equal volume of RPMI 1640 (250 μl) supplemented with 20% FCS was added to the cells. 48 h after the start of transfection (day 7), DC were washed and pulsed with 10 μg/ml of CII for 24 h. At day 8, DC were then activated with LPS/TNF-α for additional 24 h. 7 days before and/or 12 days after priming with CII, different groups of mice with 6 animals per group were i.p. injected with shRNA-transfected or control DC at a dose of 5 × 10^6 ^cells per mouse.

### RT-PCR

Total RNA was extracted from cells using Trizol (Invitrogen). 3 μg total RNA was used to synthesize the cDNA with oligdT and reverse transcriptase (Invitrogen) in 20 μl reaction volume. Primers used for the amplification of murine IL-12, IFNγ, IL-2, IL-4, IL-10 and GAPDH were as follows [37]: IL-12, 5'- CTT GCC CTC CTA AAC CAC CTC AGT-3' (forward) and 5'- CCA CCA GCA TGC CCT TGT CTA-3' (reverse); IFNγ, 5'- CAC GGC ACA GTC ATT GAA AGC CTA-3' (forward) and 5'- TGA GGC TGG ATT CCG GCA ACA GCT-3' (reverse);

IL-2, 5'- ACA TTG ACA CTT GTG CTC CGT GTC-3' (forward) and 5'- TTG AGG GCT TGT TGA GAT GAT GCT-3' (reverse); IL-4, 5'- AGC TAG TTG TCA TCC TGC TCT TCT-3' (forward) and 5'- CGA GTA ATC CAT TTG CAT GAT GCT-3' (reverse); IL-10, 5'- GAA GAC AAT AAC TGC ACC CAC TTC-3' (forward) and 5'- ATG GCC TTG TAG ACA CCT TGG TCT-3' (reverse); GAPDH, 5'-TGA TGA CAT CAA GAA GGT GGT GAA-3' (forward) and 5'-TGG GAT GGA AAT TGT GAG GGA GAT-3' (reverse).

Polymerase chain reaction (PCR) was performed in a 25 μl of reaction volume containing 0.2 μmol/L primers, 1 U Taq DNA polymerase under the following conditions: 95°C for 30 s, 58°C for 30 s, and then 72°C for 30 s (30 cycles). PCR products were visualized with ethidium bromide on 1.5% agarose gel.

### Mixed leukocyte reaction (MLR)

At day 5 of culture, bone marrow-derived DC from DBA/1 LacJ mice were transfected with IL-12 and scrambled siRNAs or mock-transfected followed by activation with LPS/TNF-α. Activated DC were irradiated (3,000 rad) and seeded in triplicate in a flat-bottom 96-well plate (Corning) for use as stimulator cells. Spleen T cells from BALB/c mice were isolated by gradient centrifugation over Ficoll-Paque (Amersham Pharmacia Biotech, Quebec) and added as responders (5 × 10^5 ^cells/well). The mixed lymphocytes were cultured at 37°C for 72 h in 200 μl of RPMI 1640 supplemented with 10% FCS, 100 U/ml of penicillin, and 100 μg/ml of streptomycin and pulsed with 1 μCi/well of ^3^H-labelled thymidine (Amersham Pharmacia Biotech) for the last 16 h of culture. Finally, cells were harvested onto glass fiber filters, and the radioactivity incorporated was quantitated using a Wallac Betaplate liquid scintillation counter (Beckman, Fullerton, CA). Results were expressed as the mean counts per min of triplicate cultures ± SEM.

### Proliferation assays

T cell proliferative responses to CII in subsequent groups of mice were measured with a standard microtiter assay. Following CII immunization, the proliferative responses could be detected for several weeks. Immune cells from either draining lymph node or spleen T cells collected from the mice treated with CII and IL-12 silenced DC or control DC, at 5 × 10^5^/well were seeded to a 96-well flat-bottom microtiter plate (Corning) in triplicates and mixed with serial dilutions of CII with concentrations ranging from 5 to 50 μg/well. Following a 72 h incubation, 1 μCi of [^3^H] thymidine (Amersham) was added to each well for 16 h. Using an automated cell harvester, the cells were collected onto glass microfiber filter, and the radioactive labeling incorporation was measured by a Wallac Betaplate liquid scintillation counter.

### Anti-CII antibody measurement

CII-specific Abs were evaluated using a standard indirect ELISA in which 500 ng of CII was absorbed to each well of a 96-well microtitre plate. Following blocking and washing steps, serial dilutions of immune mouse serum were added to the appropriate wells in duplicates and incubated overnight at 4°C. Dilutions of serum were 1:100-1:100,000. To develop the ELISA, horseradish peroxidase-conjugated goat anti-mouse IgG Fc and ortho-phenylenediamine dihydrochloride substrate buffer (Sigma) were used. The OD of each well was measured at a wavelength of 490 nm in an ELISA plate reader.

### Cytokine quantification

Mock or shRNA-transfected DC of DBA/1 LacJ origin were cultured with the allogeneic (BALB/c) T cells or alone for 48 h. The supernatants were collected and assessed for DC cytokines (IL-10 and IL-12) and T cell cytokines (IFN-γ and IL-4) by ELISA. Cytokine-specific ELISA (Endogen, Rockford, IL) was used for detecting cytokine concentrations in culture supernatants according to the manufacturer's instructions using a Benchmark Microplate Reader (Bio-Rad, Hercules, CA).

### Histology

Paws from experimental and control groups of freshly dissected mice were removed and joint tissues were immersion-fixed for 4 day in 10% (wt/vol) neutral buffered formalin in 0.15 M PBS (pH 7.4). After decalcification by immersing in Decalcifier I solution (Winnipeg, Canada) overnight and subsequent dehydration in a gradient of alcohols, tissues were rinsed in running water. The specimens were processed for paraffin embedding in paraplast (BDH, Dorset, UK) as routine procedure. Serial paraffin sections throughout the joint were cut at 5-μm thickness on a microtome, heated at 60°C for 30 min, and deparaffinized. Hydration was done by transferring the sections through the following solutions: triple to xylene for 6 min, and then for 2 min to 100% ethanol twice, 95% ethanol, and 70% ethanol. Sections were stained with H&E and mounted on glass slides.

### Intracellular cytokine staining and flow cytometry

Transfected DCs were treated with 20 ng/ml phorbol myristate acetate and stained with FITC-conjugated IL-12. Ig of the same isotype was used as controls. Flow cytometry analysis was performed in a FACScan II (Becton Dickinson, San Jose, CA- BD) system using FACSDiva software (BD).

### Statistical analysis

Data are expressed as mean ± SEM. Differences between different groups of mice were compared using the Mann-Whitney U test for nonparametric data. A *P *value less than 0.05 was considered significant.

## Results

### Silencing DC with IL-12 shRNA

Silencing of IL-12p35 on bone marrow derived DC was previously performed by our group using presynthesized siRNA [35]. Although this approach is widely used, one disadvantage is the relatively low efficacy due to consumption of the exogenously administered siRNA. An alternative approach would be to induce the cell to produce siRNA endogenously. One way of achieving this is using so-called DNA-directed RNAi (ddRNAi), which utilizes a DNA construct expressing double stranded RNA. Accordingly we transfected DC with a vector comprising a hairpin loop of siRNA (shRNA), driven by U6 promoter.

**Figure 1:**
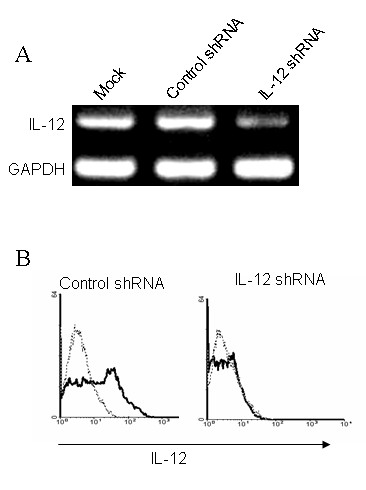
**Gene silencing of IL-12 shRNA on DC**. (***A***) Gene silencing efficacy of IL-12 shRNA was determined by RT-PCR. DC were cultured for 4 days as described in the methods. On day 5, 10^6 ^DC were transfected with 2 μg of IL-12 shRNA using GenePORTER reagent. One day after transfection, DC were harvested and mRNA detected. (***B***) Gene silencing efficacy of IL-12 shRNA was determined by flow cytometry. DC were transfected as above and collected 48 h after transfection. DC were intracellular stained with FITC labeled anti-IL-12 antibody and followed by flow cytometry analysis. The data presented one of three independent experiments.

We determined the gene silencing efficacy of IL-12 shRNA on DC by RT-PCR (Figure 1A). IL-12 shRNA reduced IL-12 expression on DC by 3-fold as compared with control. The gene silencing effect of IL-12 shRNA was further confirmed at the protein level by intracellular staining with anti-IL-12 antibody using flow cytometry (Figure 1B). These data suggest feasibility of specific gene silencing in DC using the shRNA approach.

### In vitro immune modulation by IL-12 shRNA

**Figure 2:**
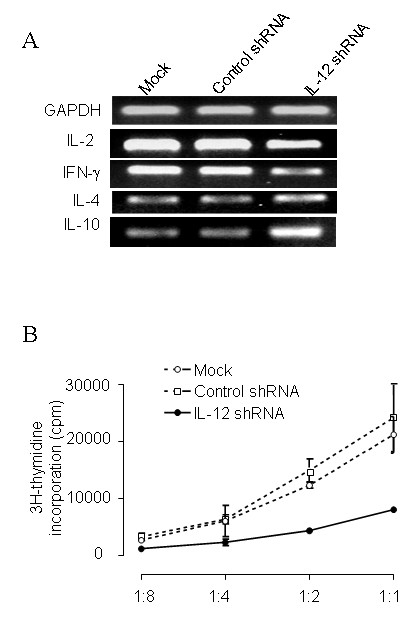
**Immune modulation by IL-12 shRNA silenced DC**. (***A***) IL-12 silenced DC promote Th2 cytokine production. Control and siRNA-treated DC were cultured with allogeneic T cells for 3 days. Co-cultured T cells were harvested and gene expression of IFNγ, IL-2, IL-4 and IL-10 were detected by RT-PCR. (***B***) DC silenced with IL-12 shRNA inhibited allogeneic T cell proliferation. DC (1 × 10^5 ^cells/well) were co-cultured with allo-T cells (5 × 10^5 ^cells/well) in 96-well plate for 72 h. 1 μCi/well of ^3^H-labelled thymidine was added to the culture for the last 16 h of culture and proliferation was assessed by scintillation counting. Results were expressed as the mean counts per min of triplicate cultures ± SEM. * = *P *< 0.05.

IL-12 is an important cytokine for the interaction of DC and T cells. IL-12 secreted by DC controls T helper cell deviation. Thus, we assessed whether silencing of IL-12p35 using shRNA would alter the ability of the DC to modulate a Th1 to Th2 shift. Using an allogeneic system, we co-cultured C57/BL6 DC, treated with IL-12 shRNA, together with responder BALB/c T cells. This resulted in a predominant Th2 cytokine profile (e.g. high IL-4, low IFN-γ), as compared to the co-culture with DC treated with control shRNA which possessed a Th1 profile (Figure 2A). We next tested the antigen presentation capacity of DC after gene silencing of IL-12 by quantifying proliferative response of the BALB/c T cells reacting against control and IL-12 silenced C57BL/6 DC. Figure 2B showed that T cell response was reduced after being stimulated by the DC in which IL-12 gene is silenced. These data suggest that IL-12 not only plays a role in production of IFN-g and IL-4, but also is involved in stimulation of T cell proliferation by allogeneic DC. Accordingly, we sought to determine whether DC modulated in this manner may be useful for modulating an immune response in vivo in an autoimmune disease model.

### Inhibition of CIA by treatment with IL-12 shRNA-transfected DC

**Figure 3:**
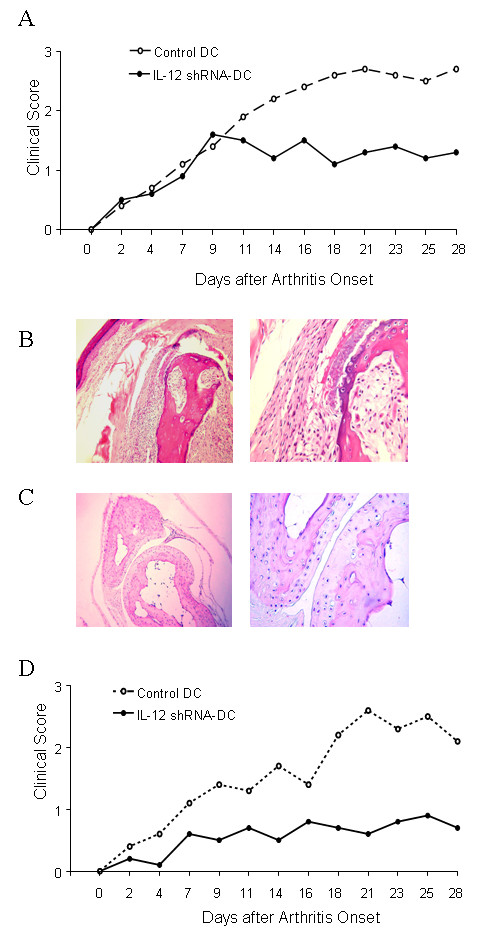
**Inhibition of clinical development of CIA in mice injected with IL-12 shRNA-transfected DC**. (***A***) Index of disease severity of joint after single DC treatment. 12 days after intradermal challenge with CII (200 μg per mouse), DBA/1 LacJ mice were treated with one i.p. injections of 5 × 10^6 ^IL-12-silenced DC pulsed with CII (10 μg/ml). Control siRNA -transfected DC pulsed with CII served as a control. All mice were i.p. boosted with the same dose of CII 21 day after priming with the antigen. The animals were observed for 4 weeks since arthritis onset. Each limb was graded on a scale from 0 to 4 and the average clinical score per affected paw was calculated. Each point denotes the score of 5 mice in each group. Results represent 1 of 3 experiments. * = *P *< 0.05 versus control DC. (***B***) Histological sections of joints from arthritis mice injected with control DC. H&E stained sagittal sections of proximal interphalangeal joints. Sections from control mice show widespread inflammation cell infiltration, mild edema and congestion. Cartilage surface became uneven due to soft bone damage. (***C***) Histological sections of joints from arthritis mice injected with IL-12 shRNA-transfected DC. The majority of sections from animals treated with single injection of IL-12-silenced DC pulsed with CII do not show monocyte infiltration, edema and congestion. Cartilage surface appears smooth. Original magnification × 100. Results represent 1 of 15 mice. (***D***) Index of disease severity of joint after two DC treatments. 7 days before and 12 days after intradermal challenge with CII (200 μg per mouse), DBA/1 LacJ mice were treated with i.p. injections of 5 × 10^6 ^IL-12-silenced DC pulsed with CII (10 μg/ml). Mock-transfected DC pulsed with CII served as a control. All mice were i.p. boosted with the same dose of CII 21 day after priming with the antigen. The animals were observed for 4 weeks since arthritis onset. Each limb was graded on a scale from 0 to 4 and the average clinical score per affected paw was calculated. Each point denotes the score of 5 mice in each group. Results represent 1 of 3 experiments. * = *P *< 0.05 versus control DC.

The CIA model of RA is an ideal system for assessment of therapeutics based on the fact that it displays similar onset, progression, and pathology to clinical RA. Importantly, the autoantigen in CIA is molecularly defined CII and T cell responses to this protein are essential for disease continuation [38]. We have previously demonstrated that silencing expression of IL-12p35 in DC leads to in vivo immune modulation of T cell response to a nominal antigen KLH [35]. In the previous study we utilized presynthesized siRNA, and we did not assess ability to inhibit immunity in a therapeutic model. In this study, we used a clinically relevant model of autoimmune arthritis and evaluated two treatment protocols. When, 12 days after priming with CII, DBA/1 mice were administered a single injection of IL-12 shRNA-transfected DC pulsed with CII, an inhibition of arthritis clinical score had been observed 11 days after arthritis onset as opposed to control-treated mice receiving DC that were not gene-silenced for IL-12 (Figure 3A). The average clinical scores per affected paw in the control group were two times higher than that in the treatment group. To confirm the therapeutic effects by IL-12 silenced DC, we further sought to examine microscopic histological differences induced by treatment of CIA mice with the IL-12-silenced and CII-pulsed DC. Accordingly, animals treated with IL-12 shRNA-transfected DC or control DC were sacrificed 4 weeks following onset of arthritis and joints were examined by serial sectioning. We observed that control mice possessed severe bone erosion, pannus formation, and synovitis (Figure 3B). A marked neutrophilic and mononuclear cell infiltration was seen. In contrast, joint histology of the IL-12-silenced DC treated mice revealed markedly attenuated morphological changes and cellular infiltration, and the preservation of normal cartilage structure (Figure 3C). The histological verification of the arthritis clinical score strongly suggests that the IL-12 silenced and CII-pulsed DCs can serve as a potent tolerogenic vaccine that might be useful for the treatment of autoimmune arthritis.

While the protocol above was therapeutic in the sense that the intervention was given after initiation of immune response, we chose to determine whether the combination of a prophylactic (i.e. 7 days before priming with CII) and the therapeutic IL-12-silenced DC injection resulted in additive or synergistic inhibition of arthritis development. Using this combination we observed a lower severity of arthritis, as compared to a single injection protocol (Figure 3D). Furthermore, the control DC group had higher average clinical score than experimental group ranged from 2-fold to more than 4-fold depending on the timepoint of assessment. This suggests that a combination of cellular vaccine strategies could lead to additive anti-arthritis effects.

### IL-12 shRNA silenced DC inhibited CII-specific T cell response in arthritic mice

**Figure 4:**
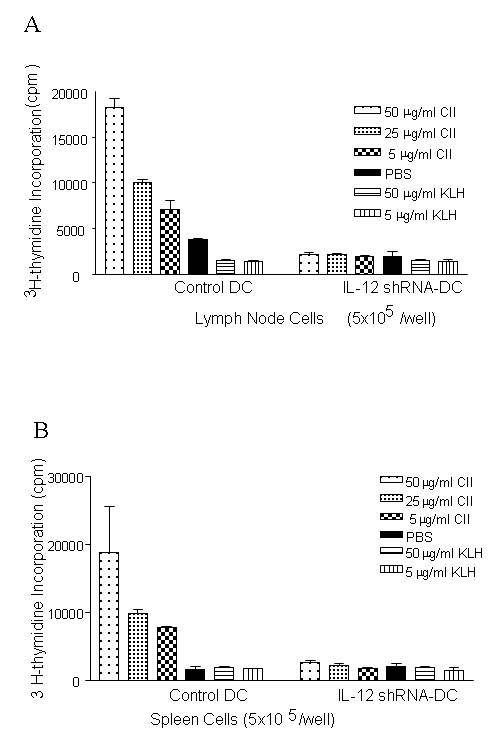
**T cell recall responses to CII in arthritis mice injected with IL-12 shRNA-transfected DC**. Experimental and control groups of mice were treated with two injections of IL-12-silenced DC as described in the legend to Figure 3. At the end of clinical assessment of CIA development, the mice were sacrificed and T cells from lymph nodes (***A***) and spleens (***B***) were isolated. A CII-specific response from different group of animals was performed by proliferation, as described in *Materials and Methods*. Lymphocytes were restimulated in vitro with different concentrations of CII, KLH or PBS alone and a ^3^H-labelled thymidine incorporation was measured. Results represent 1 of 3 similar experiments (*n *= 4 per group/experiment). * = *P *< 0.05 versus control PBS-treated DC.

It has been previously described that immune modulation by administration of IL-10-treated DC can inhibit antigen-specific recall response [39]. However, the long-term effects of immune modulation with inhibitory DC have not been reported. Here, we have measured CII-specific T cell recall responses after arthritis development in mice treated with IL-12 shRNA-transfected DC pulsed with CII. Four-weeks after arthritis onset, we extracted lymph node and spleen T cells from correspondent group of animals in 4 weeks following the first signs of arthritis. A profound suppression of response to CII was observed in T cells derived from both lymph node (Figure 4A) and spleen (Figure 4B) sources in the mice treated with IL-12 siRNA.

### Reduced threshold for inhibition of antibody response to CII in arthritis mice

**Figure 5:**
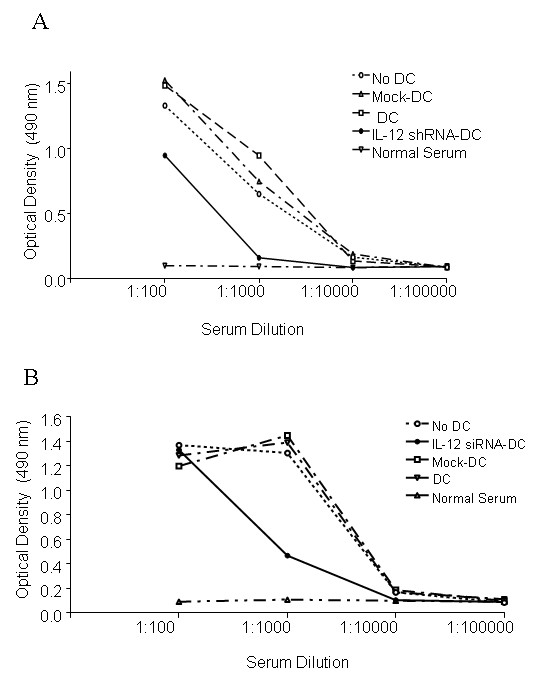
**Inhibition of CII-specific antibody production in arthritis mice following injections with IL-12 shRNA-transfected DC**. Blood was taken 4 weeks after arthritis onset from experimental and control groups of mice treated with one (***A***) or two (***B***) injections of IL-12-silenced DC, as described in the legend to the Figure 2. Serum levels of anti-CII immunoglobulin Fc were determined using ELISA. Results show average levels of antibodies expressed as OD (*n *= 4 per group/experiment). * = *P *< 0.05 versus control shRNA-transfected DC.

The importance of CII-specific antibodies in development of CIA pathology is well-known [21]. However, control of antibody responses by DC has not been previously examined in a therapeutic sense. Tolerogenic DC may directly block antibody production through inhibition of BlyS and APRIL, factors which the DC use to directly induce Ig production and class switching in B cells [40,41]. Alternatively, tolerogenic DC may indirectly prevent antibody production through the inhibition of T cell helper function. Here, we assessed antibody responses to CII in animals 4 weeks after arthritis onset. Using a titration experiment, we compared the serum levels of anti-CII Ig from control mice with CIA mice treated with single injection of IL-12-silenced/CII-pulsed DC twice, before and after CII administration. CII-specific antibody response was inhibited by both the single injection (Figure 5A) and by the two injections of the IL-12 silenced DC (Figure 5B). This indicates that the IL-12 shRNA-transfected DC represent an immunomodulatory therapeutic strategy since they are not only involved in suppression of T cell responses but also in antibody production.

## Discussion

We previously demonstrated that silencing IL-12 resulted in immune deviation and modulation in vitro and in vivo [21]. However, to date, therapeutic utilization of shRNA-transfected DC has not been performed in arthritis. In this study, we have reported that silencing the Th1-inducing cytokine IL-12 with DNA-directed RNA interference (ddRNAi) in the form of shRNA leads to a potent Th2 deviation that culminates in inhibition of CIA, the murine model of rheumatoid arthritis. This model is optimal for antigen-specific immune modulation since: 1) disease is associated with a defined antigen, CII [42]; 2) a defined cytokine response, which is known to be IL-12-dependent, leading to IFN-γ-driven activation of macrophages and synoviocytes, and is causative in the inflammatory lesions that appear [40]; and 3) the CIA model is modifiable by exogenous manipulations [43]. Our experimental protocols consisted of administering CII-pulsed IL-12-silenced DC at day -7, and/or day 12 following the first CII challenge. These protocols were used to assess both prophylactic and therapeutic effects of the tolerogenic IL-12 shRNA-transfected DC.

Mature myeloid DC possess high expression of MHC class II molecules (signal 1), costimulatory molecules (signal 2) and IL-12 (signal 3). Signals 1 and 2 stimulate T cell activation, while signal 3 polarizes T-helper (Th) differentiation. Therefore, immune modulation can be achieved through inhibition of immune molecules in DC by various blockades such as antibodies [44], fusion proteins [45,46] or antisense oligonucleotides [47], and pharmacological agents [21]. In comparison with these previously used blocking methods, silencing gene expression through ddRNAi may prove superior to conventional gene or antibody blocking approaches for the following reasons: 1) blocking efficacy is potent [48]; 2) targeting gene expression is specific to one nucleotide mismatch [49]; 3) inhibitory effects can be passed for multiple generations to daughter cells [50]; 4) *in vitro *transfection efficacy is high [35] and can be expressed in a stable manner [51]; 5) *in vivo *use may be more practical and safer than antibody approaches due to lower concentrations needed for silencing, and lack of neutralizing antibody production; 6) tissue or cell specific gene targeting is possible using specific promoter vectors [52,53] or specific antibody conjugated liposomes [27,54]; 7) simultaneously targeting multiple genes or multiple exons silencing is possible for increasing efficacy [55]. Clinical efficacy of ddRNAi has been recently demonstrated in AIDS-related lymphoma patients who received viral-vector transfected autologous CD34 cells, in which it was demonstrated that the cells and their progeny expressed the shRNA for at least two years from a single ex vivo transfection treatment [56].

In contrast to other methods of DC modulation, induction of RNAi in this cell population offers an approach to specifically modify the immune-regulating abilities of DC. In our first description of shRNA-manipulated DC, we demonstrated that silencing of IL-12p35 is sufficient to induce a DC population that stimulates Th2 immune responses in vitro and in vivo [35]. Since DC can be pulsed with antigenic peptides or mRNA ex vivo, the shRNA-modification of this cell type offers the ability to generate vaccines not only for stimulation but also for inhibition of immunity. Subsequent to our initial study, two other groups have reported utilization of RNAi in DC. Laderach et al [57] reported specific and efficient silencing the NF-κB gene in human monocyte-derived DC using siRNA transfected via electroporation. This study was particularly interesting since it clearly demonstrated the ability of siRNA to study the specific functions of subunits comprising multimeric transcription factors [57]. Silencing of DC cell lines using shRNA was used by Wong et al to silence the Plexin A1 gene, demonstrating that this neuronal-specific protein is critical in DC-T cell interactions [58]. More recently, we induced transplant tolerance through gene silencing of RelB [59]. These reports indicate the utility of RNAi for immunological and molecular investigations of DC. Despite the success of silencing DC by siRNA, several key issues of gene silencing in DC remains undetermined, such as siRNA delivery methods, persistence of silencing efficacy, and multiple gene silencing. We demonstrate that RNA interference can be accomplished in DC either by transient delivery of presynthesized siRNA or by transfection of plasmid encoding shRNA. Utilizing shRNA, the gene silencing efficacy can last at least up to the end point of DC culture [60].

## Conclusion

These results serve as a proof-of-principle study, illustrating that DNA-directed RNA interference in the form of shRNA can be used efficiently, and rapidly for production of tailor-made tolerogenic DC that are primed with specific antigen for a cellular "tolerogenic vaccine". With the identification of a plethora of novel immune stimulatory genes, it raises the possibility of using ddRNAi gene-silenced tolerogenic DC for individualized therapy of autoimmune diseases.

## Abbreviations

siRNA: Small interfering RNA; shRNA: Short hairpin RNA; ddRNAi: DNA-directed RNA interference; CII: Type II collagen; CIA: Collagen-induced arthritis; DCs: Dendritic cells; Treg: Regulatory T cell.

## Competing interests

The authors declare that they have no competing interests.

## Authors' contributions

RL, XiZ, IP, XuZ, HW, MS, DC, LS carried out the experiments, WM, RI, PF, RN, DK participated in the project design, coordination the experiments, and helped to draft the manuscript. All authors read and approved the final manuscript.
